# Phytochemical characterization by GC-MS and *in vitro* evaluation of antiproliferative and antimigratory studies of *Leucas aspera* leaf extracts on MDA-MB-231 cell line

**DOI:** 10.5114/bta.2024.135642

**Published:** 2024-03-29

**Authors:** Mahaboob Begum S.M. Fazeela, Megasri Sankarram

**Affiliations:** B.S. Abdur Rahman Crescent Institute of Science and Technology, Vandalur, Tamil Nadu, India

**Keywords:** cytotoxic activity, MDA-MB-231, breast cancer, *Leucas aspera*, anti-migratory efficiency

## Abstract

Breast cancer is the most recurrently identified and one of women’s prominent causes of death. Currently, researchers have turned their focus on natural chemicals from synthetic chemicals due to their environmental, economic, and health benefits. Considering this, the medicinal plant *Leucas aspera* was chosen for the current study. The aim of this study was to isolate and characterize secondary metabolites from *L. aspera* and determine the antiproliferative and antimigratory activities in the MDA-MB-231 cell line under *in vitro* conditions. Phytochemicals from *L. aspera* were isolated through sequential extraction using hexane, dichloromethane, and ethyl acetate. These extracts were qualitatively screened, subjected to FT-IR, and analyzed using GC-MS. The antiproliferative activity was determined through the MTT assay. Scratch assay was utilized to determine the antimigratory activity of the plant extracts. The phytochemical analysis revealed the presence of steroids, alkaloids, phenols, flavonoids, galactose, tannins, saponins, and amino acids in the extracts. The results of the cell viability assay indicated that the crude dichloromethane and ethyl acetate extracts inhibited cell proliferation, with inhibitory concentrations of 5 and 3 μg/ml, respectively. In contrast, the crude hexane extract did not exhibit any cytotoxicity. Furthermore, the scratch assay results showed that the plant extracts had cell migration inhibitory properties. The outcomes of the current study conclude that *L. aspera* possesses active therapeutic agents with strong anticancer potential, effectively impeding the proliferation and invasion of MDA-MB-231. Further studies are needed to identify the potential active agents that contribute to these activities.

## Introduction

The human body is made up of numerous cells. Usually, when cells mature and get damaged, they need new cells to replace them. This replacement is done by the process of cell division where a cell grows and multiplies. However, when this process becomes deregulated, abnormal cell growth and multiplication may leading to the formation of tumors. These tumors can be either benign or cancerous (malignant). Cancerous tumors undergo a process called metastasis, where the tumors metastasize neighboring tissues and can migrate to distant places to form secondary tumors (World Health Organization, 2021).

Breast cancer is considered as second most common cancer among women. It originates in the epithelial cells lining the ducts and lobules of the breast’s glandular tissue (National Cancer Institute, 2021). According to the World Health Organization, in 2020, approximately 2.3 million new cases of breast cancer were diagnosed globally, resulting in 685,000 reported breast cancer deaths were reported worldwide (World Health Organization, 2021). In India, 26.3% of the female population was affected by breast cancer in 2020, with 90 408 deaths among 178 361 new cases (International Agency for Research on Cancer, 2021). Globally, the incidence of breast cancer is increasing rapidly making females more vulnerable. It accounts for 23% of total cancer cases among females (Global Cancer Observatory, 2021). In many developed countries like the United States, United Kingdom, Australia, and Canada, the mortality rate of cancer has been declining with advancements and refinement in diagnostic techniques and treatment methods, despite the fact breast cancer seems to be the leading cause of cancer death in females (National Cancer Institute, 2021). Among the various types of breast cancer, triple-negative breast cancer (TNBC) accounts for 10–20% of cases. TNBC is a heterogenous tumor where the cancer cells lack estrogen receptor (ER), progesterone receptor (PR), and human epidermal growth factor receptor 2 gene (HER2) amplification (Kumar and Aggarwal, 2016).

Normal cell division and DNA segregation are regulated by cell cycle checkpoints. The control of cell cycle checkpoints and DNA repair is done by BReast CAncer gene 1 (BRCA1) which acts as a tumor suppressor gene. In breast cancer cells this BRCA1 gene is mutated therefore it cannot repress the estrogen receptor α (ER-α) resulting in the development of TNBC (Sengodan et al., 2018). This mutation indirectly promotes the carcinogenesis of other genes by increasing their mutation rate (Lin et al., 2022).

Treatment options for TNBC, are limited, as hormonal therapy is ineffective due to the absence of ER, PR, and HER2 receptors. So, surgery and chemotherapy are the only ways to treat TNBC (Wahba and El-Hadaad, 2015). Despite being more aggressive, the surgical decision relies on the clinicopathological variables and patient’ preferences. Traditionally radiation therapy has been given to TNBC which is followed by mastectomy or conservative breast surgery (CBS). It is said that CBS followed by radiation-based therapy is equivalent to mastectomy, now this procedure remains a controversy (Al-Mahmood et al., 2018). Chemotherapy is best suggested to treat TNBC to target the DNA repair complex, p53, and cell proliferation (Chen et al., 2021). Adjuvant-based chemotherapy can be more effective in signaling the DNA repair process, but this has not been confirmed in stage III studies (Polley et al., 2021). Moreover, chemotherapy has also shown a few side effects which include nausea, vomiting, fatigue, hair loss, bruising, and bleeding (Centre for Disease Control and Prevention, 2022). Since TNBC is a very uncommon subtype of breast cancer, drugs for standard treatment are not yet available (American Cancer Society, 2022).

Due to many challenges associated with cancer treatment, researchers have redirected their attention towards natural therapies, exploring plant-derived products in the hope of mitigating adverse side effects. Despite the promising anticancer properties, exhibited by certain plant-derived products in preclinical studies, their evaluation in human subjects is still pending (Desai et al., 2008). From ancient civilizations, indigenous plants have been used as alternatives to treat diseases. Recent studies showed that nearly 65% of anticancer drugs are derived from natural compounds that were isolated from plants (Mbele et al., 2017).

One such medicinal plant under scrutiny is *Leucas aspera* (Family: *Lamiaceae*, Order: *Tubiflorae* ), commonly known as “Thumbai”. This annual, branched herb, reaching heights of 15–60 cm, is widely distributed throughout India (Prajapati, 2010). The plant has been used in traditional medicinal systems such as Ayurveda and Siddha. *L. aspera* has been previously studied for its diverse medicinal properties, including antihistaminic, antipyretic, antifungal, antimicrobial, and antioxidant attributes (Nirmala and Kanchana, 2018; Vasudha et al., 2019; Kumar and James 2020; Mishra et al., 2022). With its extensive medicinal properties, *L. aspera* is believed to have various active phytochemicals with anticancer properties, as suggested by prior studies (Latha et al., 2013).

Despite these indications, the effect of bioactive molecules from *L.aspera* and their activity has not yet been studied in TNBC cell line MDA-MB-231. Our current study focused on discovering the active therapeutic agents present in *L. aspera* and assessing their cytotoxic and antimigratory activities on the MDA-MB-231 cell line under *in vitro* conditions.

## Materials and methods

### Collection of plant material

The powdered *L. aspera* leaves were obtained from Annai Aravindh Herbals in Chennai, India. This identical powdered leaf form was used for both identifying phytochemical components and conducting *in vitro* evaluation of the antiproliferative and antimigratory properties of *L. aspera*.

### Extraction of phytochemicals from Leucas aspera

Extraction of the plant’s phytochemicals was carried out by sequential method as outlined in the published protocol (Begum et al., 2018). Thirty grams of plant leaf powder underwent extraction with 100 ml of solvents in increasing order of polarity: hexane, followed by dichloromethane, and finally, ethyl acetate. The extraction process took place in an orbital shaker over 72 h. Subsequently, the extract was filtered using Whatman filter paper No: 1 (Sigma – Aldrich), and the filtrate was subjected to evaporation in a rotary evaporator. The resulting dried extracts were stored at 4˚C in storage vials for future studies. This extraction procedure was performed twice, and the extracts were collected following the aforementioned steps.

### Phytochemical analysis of Leucas aspera crude extracts

Analysis of phytoconstituents in the crude plant extracts was performed as per standard protocols (Sharma et al., 2021) for alkaloids, flavonoids, tannins, saponins, glycosides, and terpenoids.

### Fourier Transform Infrared (FT-IR) spectrometry

To determine various bioactive functional groups within the extracts, FT-IR spectrometry analysis was performed. The dried crude extracts of hexane, dichloromethane, and ethyl acetate of *L. aspera* were dissolved in methanol (1 mg/ml) and subjected to FT-IR analysis which was based on attenuated total reflection (ATR) (Kumar and Ramaswamy, 2014). This method is based on internal reflection within an optical sensor material. The FT-IR spectrum intensity depends on the number of reflections, the number of molecules of interest and their absorptivity, and the depth of penetration into the sample. The functional group present in each extract was studied using the IR spectrum obtained with 4/cm resolution after 25 scans.

### GC-MS analysis of Leucas aspera

The crude hexane, dichloromethane, and ethyl acetate extracts of *L. aspera* were analyzed in GCMSQP2010 Plus to identify the functional groups present in the extracts. The column temperature was set 45˚C, the injection temperature at 280˚C, the ion source temperature at 250˚C, and the interface temperature at 260˚C. The pressure was maintained at 51.5 kPa, the total flow volume at 54 ml/min, and the solvent cut time was set to 2 min (Konappa et al., 2020). The sample, dissolved in methanol (1 : 10 split ratio), was injected with a volume of 1 μl. Utilizing the mass spectrum generated, a NIST library search was conducted to identify the phytocompounds present in the extracts.

### Cell line and cell culture

MDA-MB-231 cells were generously provided by Dr. P. Ashok Kumar from the School of Life Sciences at B.S. Abdur Rahman Crescent Institute of Science and Technology, Chennai, India, and were used in the study. The cells were subcultured in DMEM (high glucose) (HiMedia, USA), supplemented with 10% fetal bovine serum (HiMedia, USA), and 1% antibiotic cocktail (HiMedia, USA). Cultivation of the cells took place in an atmosphere with 5% carbon dioxide at 37˚C. Actively dividing cells were used for further analysis.

### Evaluation of cell cytotoxicity of Leucas aspera extracts

The cytotoxic effects of *L. aspera* extracts were evaluated using the 3-(4,5-dimethylthiazol-2-yl)-2,5-diphenyltetrazolium bromide (MTT) assay, which involves the reduction of MTT to generate formazan crystals (Dubey et al., 2022). MDA-MB-231 cells were subcultured in DMEM (high glucose), supplemented with 10% FBS and 1% antibiotic cocktail (penicillin G, streptomycin, and fungizone) (HiMedia, USA), and seeded at a density of 1 × 10^5^ cells/well in a 96-well tissue culture plate. The cells were then treated with increasing concentrations of hexane, dichloromethane, and ethyl acetate extracts of *L. aspera* in triplicate, followed by incubation for 24 h at 37˚C. Subsequently, MTT reagent was added to each well and incubated for 2 h at 37˚C. After 2 h, the formazan crystals were solubilized in isopropanol and incubated at room temperature for 20 min. Untreated cells served as controls. Absorbance was measured at 570 nm using a multimode plate reader. The experiment was conducted in triplicate, and the percentage of cell viability was calculated using the formula:


$$\text{\% Cell viability =}\frac{\text{Average absorbance of treated cells}}{\text{Average absorbance of control}}\times 100$$

Doxorubicin served as the standard reference. Subsequently, to understand alterations in cellular morphology, the cells were exposed to the inhibitory concentration (IC_50_) of both dichloromethane (5 μg/ml) and ethyl acetate (3 μg/ml) extracts of *L. aspera* and incubated for 24 h. The morphological changes in treated and control cells were observed under a microscope at 0 and 24 h. The experiment was conducted in triplicate and repeated three times. As the hexane extract did not exhibit toxicity in MDA-MB 231 cells, it was excluded from further studies.

### Scratch assay

The anti-invasion effects of *L. aspera* extract on cell migration were assessed using the migration/scratch assay (Hobani, 2022). Actively dividing MDA-MB-231 cells (1 × 10^5^ cells/well) were subcultured in 6-well tissue culture plates until reaching 70% confluence. A wound was created on the cell monolayer by scratching with a 200 μl micropipette tip. Detached cells were washed and removed with PBS. Subsequently, the cells were treated with serum-free DMEM (high glucose) medium containing dichloromethane (3 μg/ml) and ethyl acetate extracts (2 μg/ml) of *L. aspera* for 24 h. Cell migration within the wound was determined through imaging at 0 and 24 h. Cells without any treatment served as the control. The experiment was conducted in triplicate, and the results were recorded.


$$\text{\% Cell migration =}\frac{\text{Number cells migrated in extract treated well}}{\text{Number of cells migrated in control}}\times 100$$

### Statistical analysis

All the experiments were carried out thrice in triplicate. The values expressed are mean ± SD. IBN SPSS 23 Student *t*-test was used to determine the level of significance.

## Results and discussion

The extracts obtained through sequential extraction with different solvents, following the polarity order: hexane < dichloromethane < ethyl acetate, resulted in dry weights of 0.40, 0.36, and 0.06 g, respectively, after evaporation. Extraction plays a crucial role in separating soluble plant metabolites, with separation occurring based on the solubility of compounds, as solvents of increasing polarity were utilized. Earlier studies have demonstrated that sequential extraction with solvents of varying polarity enhances the extraction of phytocompounds (John et al., 2018). Phytochemical screening of crude extracts of *L. aspera* indicated the presence or absence of various compounds such as alkaloids, saponins, flavonoids, glycosides, tannins, and terpenoids (Table 1). The order of active phytochemicals was hexane < dichloromethane < ethyl acetate extracts. The results showed that with increasing polarity, the number of extracted phytochemicals also increased. For instance, the hexane extract lacked saponins, flavonoids, and proteins, but these phytochemicals were observed in both dichloromethane and ethyl acetate extracts of *L. aspera* as polarity increased. These findings can be compared with previous research where methanol and hexane extracts of Leucas aspera were reported to contain saponins, flavonoids, tannins, alkaloids, carbohydrates, and cholesterol (Vasudha et al., 2019). However, the current study did not reveal the presence of carbohydrates and cholesterol in the extracts, possibly due to differences in solvent polarity and extraction methods.

**Table 1:** Qualitative analysis of the phytochemicals of *Leucas aspera*

Phytochemical constituents	Hexane	Dichloromethane	Ethyl acetate
Alkaloids	+	+	+
Saponins	-	+	+
Flavonoids	-	+	+
Glycosides	+	+	+
Tannins	+	+	+
Terpenoids	+	+	+
Carbohydrates	-	-	-
Protein	-	+	+
Resins	-	-	-

(+) – presence, (–) – absence

Previous reports have highlighted the high anticancer effects of flavonoids, including cell cycle arrest, induction of apoptosis, and the suppression of cell proliferation and migration (Kopustinskiene et al., 2020). Alkaloids, on the other hand, are considered natural antioxidants and may act as cancer chemopreventive agents (Cui et al., 2006; Vasudha et al., 2019). Saponins and tannins, identified in the extracts of *L. aspera*, are highly active phytocompounds. Saponins hinder the DNA replication process, thereby inhibiting cancer cell proliferation and demonstrating antiproliferative activity. Tannins also exhibit high apoptotic activity (Yildirim and Kutlu, 2015). These diverse phytoconstituents present in the extracts of *L. aspera* may contribute to its biological activity.

**Fig. 1:**
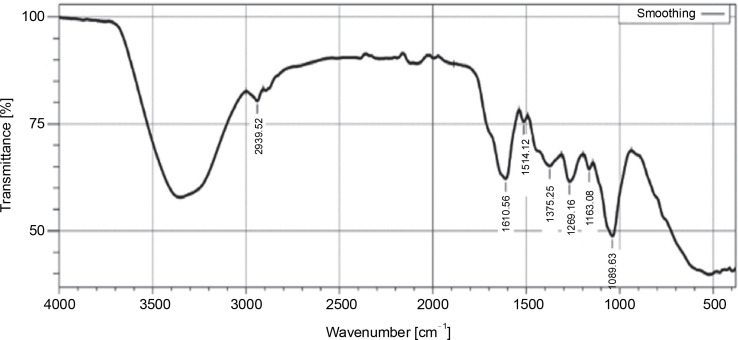
FTIR profile of hexane extract of *Leucas aspera*

The FT-IR analysis of the hexane extract of *L. aspera* revealed distinctive peaks indicative of various functional groups: C–H stretching of alkane (2914.44/cm), O–H stretching of alcohol (2850.79/cm), C=O stretching of aliphatic ketone (1708.93/cm), C–H bending of alkane-methyl group (1452.40/cm), CN stretching of aromatic amine (1367.53/cm), CO stretching of ester (1172.72/cm), C=C bending of alkene (970.19/cm), and C=C bending of alkene (725.56/cm) in the extract. The FTIR spectra are given in (Fig. 1) and the functional groups identified are given in Table 2.

**Table 2:** Functional groups identified with FT-IR analysis in hexane extract of *L. aspera*

S. No.	Peak	Compound class	Group
1	725.56	alkene	C=C bending
2	970.19	alkene	C=C bending
3	1172.72	esther	C=O stretching
4	1367.53	aromatic amine	C–N stretching
5	1452.40	alkane-methyl group	C–H bending
6	1708.93	aliphatic ketone	C=O stretching
7	2850.79	alcohol	O–H stretching
8	2914.44	alkane	C–H stretching

**Fig. 2:**
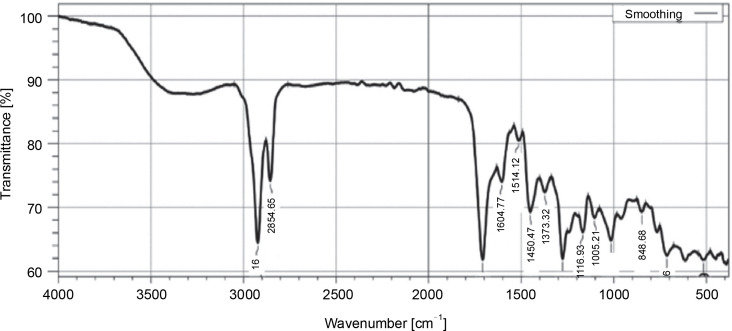
FTIR profile of dichloromethane extract of *L. aspera*

The FT-IR analysis of the dichloromethane extract of *L. aspera*, revealed characteristic peaks representing different functional groups: C–O stretching of ester (1166.93/cm), C–O stretching of aromatic ester (1276.88/cm), C–N stretching of aromatic amine (1373.32/cm), O–H stretching of alcohol (2854.65/cm), O–H stretching of carboxylic acid (2922.16/cm), C=O stretching of aliphatic ketone (1708.93/cm), N–H bending of amine (1604.77/cm), N–O stretching of nitro compounds (1514.12/cm), C–H bending of benzene derivatives (713.66/cm), and C=C bending of trisubstituted alkanes (848.68/cm) was revealed (Fig. 2, Table 3).

**Table 3:** Functional groups identified with FT-IR analysis in dichloromethane extract of *L. aspera*

S. No.	Peak	Compound class	Group
1	713.66	benzene derivative	C–H bending
2	848.68	alkane-trisubstituted	C=C bending
3	1105.21	amine	C–N stretching
4	1166.93	ester	C–O stretching
5	1276.88	aromatic ester	C–O stretching
6	1373.32	aromatic amine	C–N stretching
7	1450.47	alkane-methyl group	C–H bending
8	1514.12	nitro compound	N–O stretching
9	1604.77	amine	N–H bending
10	1708.93	aliphatic ketone	C=O stretching
11	2854.65	alcohol	O–H stretching
12	2922.16	carboxylic acid	O–H stretching

**Fig. 3:**
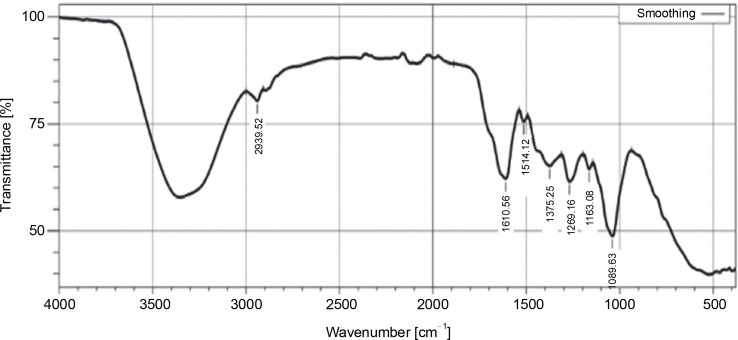
FTIR profile of ethyl acetate extract of *L. aspera*

The FT-IR analysis of the ethyl acetate extract of *L. aspera* exhibited characteristic peaks corresponding to various functional groups: S=O stretching of sulfoxide (1039.63/cm), C–O stretching of aliphatic ether (1163.08/cm), C–O stretching of alkyl aryl ether (1269.16/cm), O–H stretching of phenol (1375.25/cm), NO stretching of nitro compound (1514.12/cm), C=C stretching of α, β-unsaturated ketone (1610.56/cm), and CH stretching of alkane (2939.52/cm) was confirmed (Fig. 3, Table 4). In previous studies, FT-IR analysis revealed that the alkaloids exhibited NH stretching, phenols and flavonoids had OH stretching (Devi and Battu, 2019), saponins had C=O stretching (Bajad and Pardeshi, 2016), tannins possess C=C stretching (Marques, 2021). The outcomes of the current study revealed that dichloromethane and ethyl acetate extracts of *L. aspera* have alkaloids, flavonoids, phenols, saponins, and tannins, whereas the hexane extract of *L. aspera* showed the presence of alkaloids and tannins and these are in correlation with previous studies of Das et al. (2011), Vasudha et al. (2019).

**Table 4:** Functional groups identified using FT-IR analysis of ethyl acetate extract of *L. aspera*

S. No.	Peak	Compound class	Group
1	1039.63	sulfoxide	S=O stretching
2	1163.08	aliphatic ether	C–O stretching
3	1269.16	alkyl aryl ether	C–O stretching
4	1375.25	phenol	O–H stretching
5	1514.12	nitro compound	N–O stretching
6	1610.56	unsaturated ketone	C=C stretching of α, β-unsaturated ketone
7	2939.52	alkane	C–H stretching

**Fig. 4:**
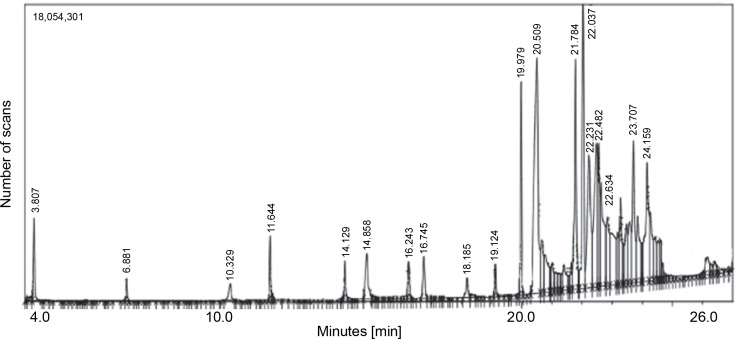
GC-MS analysis of *L. aspera* hexane extract; the GC-MS analysis revealed the presence of various compound at different retention time

**Fig. 5:**
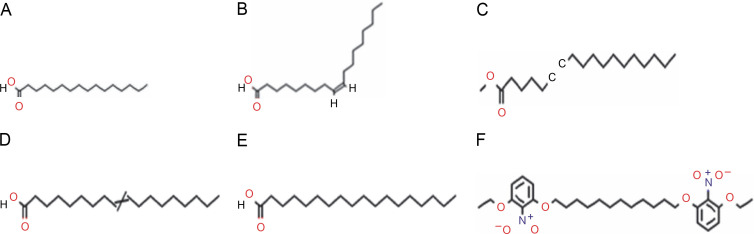
Structures of compounds identified in hexane extract of *L. aspera*. A) n-hexadecanoic acid, B) 9-octadecenoic acid, methyl ester, C) 6-octadecynoic acid, D) octadec-9-enoic acid, E) octadecanoic acid, F) dodecane, 1,12- di (2-nitro 3-ethoxyphenoxy)

The GC-MS chromatogram of the hexane extract of *L. aspera* (Fig. 4) showed 19 peaks representing different phytoconstituents. Identification of these phytoconstituents was achieved by comparing their retention time (RT), peak area, peak height, and mass fragmentation spectra with known compounds in the National Institute of Standards and Technology (NIST) library (Table 5). Among these 19 compounds, dodecane,1,12-di (2-nitro 3-ethoxyphenoxy) emerged as the most abundant, constituting 24.159% of the peak area. Other major compounds included octadecanoic acid (22.634% peak area), octadic-9-enoic acid (22.231% peak area), 6-octadecynoic acid (22.037% peak area), 9-octadecenoic acid, methyl ester (21.784% peak area), and nhexadecanoic acid (20.509% peak area). The structures of the identified compounds are illustrated in Figure 5.

**Table 5:** GC-MS analysis of *L. aspera* hexane extract

No.	Retention time [min]	PubChem CID	Name of compound	Molecular formula	Molecular weight
1	20.509	985	n-hexadecanoic acid	C_16_H_32_O_2_	256
2	21.78	5364509	9-octadecenoic acid, methyl ester	C_19_H_36_O_2_	296
3	22.037	556781	6-octadecynoic acid	C_19_H_34_O_2_	294
4	22.231	965	octadec-9-enoic acid	C_18_H_34_O_2_	282
5	22.482	5281	octadecanoic acid	C_18_H_36_O_2_	284
6	24.159	543867	dodecane,1,12-di(2-nitro3-ethoxyphenoxy)	C_2_H_4_N_2_O_8_	532

**Fig. 6:**
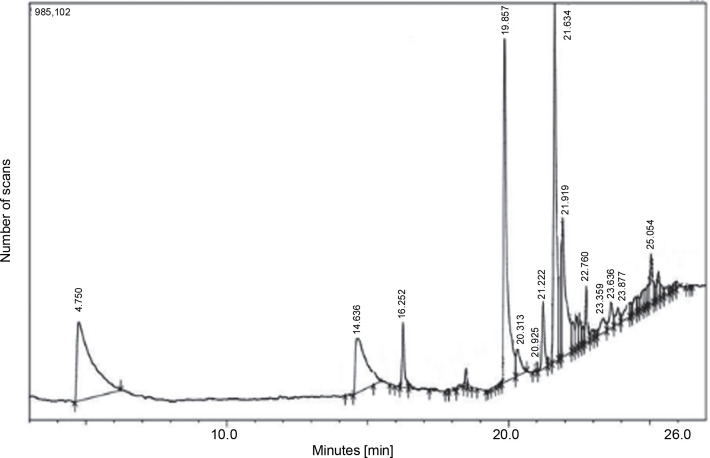
GC-MS analysis of *L. aspera* dichloromethane extract; the GC-MS analysis revealed the presence of various compound at different retention time

Similarly, the GC-MS chromatogram of the dichloromethane extract of *L. aspera* showed 13 compounds (Fig. 6). The most abundant compounds included n-hexadecanoic acid (16.4% peak area, oleic acid (14.25% peak area), 17-octadecen-14-yn-1-ol (8.48% peak area), methylparaben (7.95% peak area), 6-octadecenoic acid (16.4% peak area), and 6-octadecenoic acid methyl ester, (Z) (2.65% peak area), and bicyclo [2.2.1] heptan-2-one, 5-bromo-1,7,7-trimethyl (2.22% peak area) (Table 6). The structures of the identified compounds are presented in Supplementary Figure S1.

**Table 6:** GC-MS analysis of *L. aspera* dichloromethane extract

No.	Retention time [min]	PubChem CID	Name of compound	Molecular formula	Molecular weight
1	14.635	7456	methylparaben	^C^_8_^H^_8_^O^_3_	152
2	16.252	572870	bicyclo[2.2.1]heptan-2-one, 5-bromo-1,7,7-trimethyl-	C_10_H_15_BrO	230
3	19.857	985	n-hexadecanoic acid	^C^_16_^H^_32_^O^_2_	256
4	21.22	5366845	6-octadecenoic acid, methyl ester, (Z)-	^C^_19_^H^_36_^O^_2_	296
5	21.634	445639	oleic acid	^C^_18_^H^_34_^O^_2_	282
6	21.919	28963	17-octadecen-14-yn-1-ol	^C^_18_^H^_32_^O^	264
7	22.760	573804	2,2,4a,7a-tetramethyldecahydro-1H-cyclobuta[e]inden-5-ol	^C^_15_^H^_26_^O^	222
8	25.054	440195	flavone 4'-OH,5-OH,7-di-o-glucoside	^C^_27_^H^_30_^O^_15_	594
9	23.636	5366009	2,6,10,15,19,23-hexamethyl-tetracosa-2,10,14,18,22-pentaene-6,7-diol	^C^_30_^H^_52_^O^_2_	444
10	18.485	10446	neophytadiene	^C^_20_^H^_38_	278
11	20.925	5281	octadecanoic acid	^C^_18_^H^_36_^O^_2_	284
12	18.280	91730075	(z)-valerenyl acetate	^C^_17_^H^_26_^O^_2_	262
13	26.358	11172	cyclononasiloxane	^C^_18_^H^_54_^O^_9_^Si^9	666

**Fig. 7:**
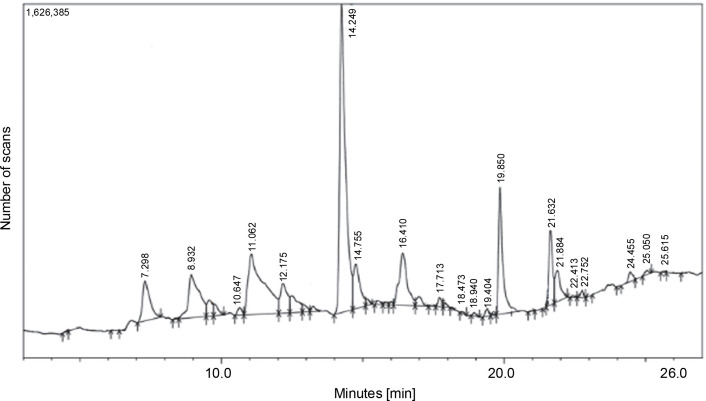
GC-MS analysis of *L. aspera* ethyl acetate extract; the GC-MS analysis revealed the presence of various compounds at different retention time

Furthermore, the GC-MS chromatogram of the ethyl acetate extract of *L. aspera* displayed 19 compounds (Fig. 7). The most abundant compounds identified in this extract were methylparaben (28.74% peak area), phenol, 4-ethenyl-, acetate (14.78% peak area), n-hexadecanoic acid (8.57% peak area), 1,3,4,5-tetrahydroxycyclohexan (6.66% peak area), oleic acid (4.49% peak area), and cyclohex-2-enyl acetic acid (2.74% peak area) (Table 7). The structures of the identified compounds are depicted in Supplementary Figure S2.

**Table 7:** GC-MS analysis of *L. aspera* ethyl acetate extract

S.No	Retention time [min]	PubChem CID	Name of compound	Molecular formula	Molecular weight
1	10.647	565060	8-azabicyclo[3.2.1]oct-6-en-3-one, 8-methyl	C_8_H_11_NO	137
2	11.062	76731	phenol, 4-ethenyl-, acetate	^C^_10_^H^_12_^O^_2_	162
3	12.175	332	2-methoxy-4-vinyl phenol	^C^_9_^H^_10_^O^_2_	150
4	14.249	7456	methylparaben	^C^_8_^H^_8_^O^_3_	152
5	14.755	11947765	beta-D-glucopyranose, 1,6-anhydro	^C^_6_^H^_10_^O^_5_	162
6	16.410	1064	1,3,4,5-tetrahydroxycyclohexanecarboxylic acid	^C^_7_^H^_12_^O^_6_	192
7	17.713	11005	tetradecanoic acid	^C^_14_^H^_28_^O^_2_	228
8	18.473	10446	neophytadiene	^C^_20_^H^_38_	278
9	18.940	534620	2-octylcyclopropene-1-heptanol	^C^_18_^H^_34_^O^	266
10	19.404	8181	hexadecanoic acid, methyl ester	^C^_17_^H^_34_^O^_2_	270
11	19.850	985	n-hexadecanoic acid	^C^_16_^H^_32_^O^_2_	256
12	21.632	445639	oleic acid	^C^_18_^H^_34_^O^_2_	282
13	21.884	227350	(cyclohex-2-enyl)acetic acid	^C^_8_^H^_12_^O^_2_	140
14	22.413	86056	bicyclo[4.1.0]heptan-3-ol, 3,7,7-trimethyl-	^C^_10_^H^_18_^O^	154
15	22.752	538938	spiro[4.5]decan-7-one, 1,8-dimethyl-8,9-epoxy-4-isopropyl-	^C^_15_^H^_24_^O^_2_	236
16	24.455	21139795	androstan-17-ol, 2,3-epoxy-, (2.alpha.,3.alpha.,5.alpha.,17.beta.)	^C^_19_^H^_30_^O^_2_	290
17	7.298	753	glycerin	^C^_3_^H^_8_^O^_3_	92
18	8.932	6993818	1,2,3-propanetriol, 1-acetate	^C^_5_^H^_10_^O^_4_	134
19	25.615	91713331	adipic acid, dec-4-enyl isobutyl ester	^C^_20_^H^_36_^O^_4_	340

Certain phytochemical compounds, including methylparaben, neophytadiene, n-hexadecanoic acid and oleic acid, were identified in both the dichloromethane and ethyl acetate extracts of *L. aspera*. Neophytadiene has been previously associated with antioxidant, antimicrobial, antifungal, and anti-inflammatory properties (Raman et al., 2012). Oleic acid , known for its apoptotic activity, activates the expression of tumor suppressor genes, particularly p27, p21, and p53, especially in human esophageal cells (Moon et al., 2014).

Flavone 4′-OH,5-OH,7-di-o-glucoside, found in previous studies, has been linked to antioxidant and antimicrobial properties. For instance, El-Sharkawy et al. reported that this flavone, identified in the extract of *Launaea nudicaulis*, exhibited significant free radical scavenging activity (IC_50_ 7.1g/l). Similarly, the extract of *L. nudicaulis* also exhibited good antimicrobial activity against both gram-positive and gram-negative bacteria, with a zone of inhibition ranging from 6.7 to 10.5 mm (El-Sharkawy et al., 2014). Octadecanoic acid is reputed to possess antibacterial, anticancer, and anti-histaminic properties (Khan and Javaid, 2022). Manivannan et al. (2017) have shown that octadecanoic acid induces apoptosis through DNA fragmentation in MCF7 and HePG2 cell lines.

Similar compounds identified in the current study have been reported in extracts of quinoa, known for its antifungal, antibacterial, antioxidant, and anticancer properties (Khan et al., 2021). Therefore, the findings from the current study, suggest that *L. aspera* possesses active phytochemical compounds with antioxidant, anticancer, and apoptotic activities.

**Fig. 8:**
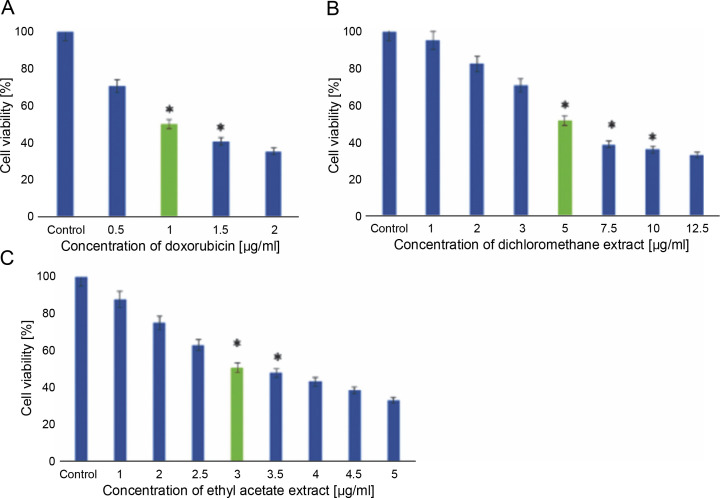
*In vitro* anticancer activity of *L. aspera* in MDA-MB-231 cells; the cytotoxicity analysis revealed IC50 as A) 1 μg/ml for doxorubicin, B) 5 μg/ml for Dichloromethane extract and C) 3 μg/ml for Ethyl acetate extract; values are shown as mean ± SD as the experiment was conducted in triplicates; *P < 0.05 are considered significant

MDA-MB-231 cells were subjected to treatment with hexane, dichloromethane, and ethyl acetate extracts of *L. aspera*, and their cytotoxic activity was evaluated through MTT assay. The outcomes indicated that the hexane extract did not impact the viability of MDA-MB-231 cells (results not shown). In contrast, dichloromethane treatment induced cell death with an IC_50_ of 5 μg/ml, and ethyl acetate treatment induced cell death with an IC_50_ of 3 μg/ml in MDA-MB-231 cells. Doxorubicin, used as a standard, exhibited an IC_50_ value of 1 μg/ml. Cells not exposed to any extract served as the control. The results of the cytotoxicity assay are presented in Figure 8.

**Fig. 9:**
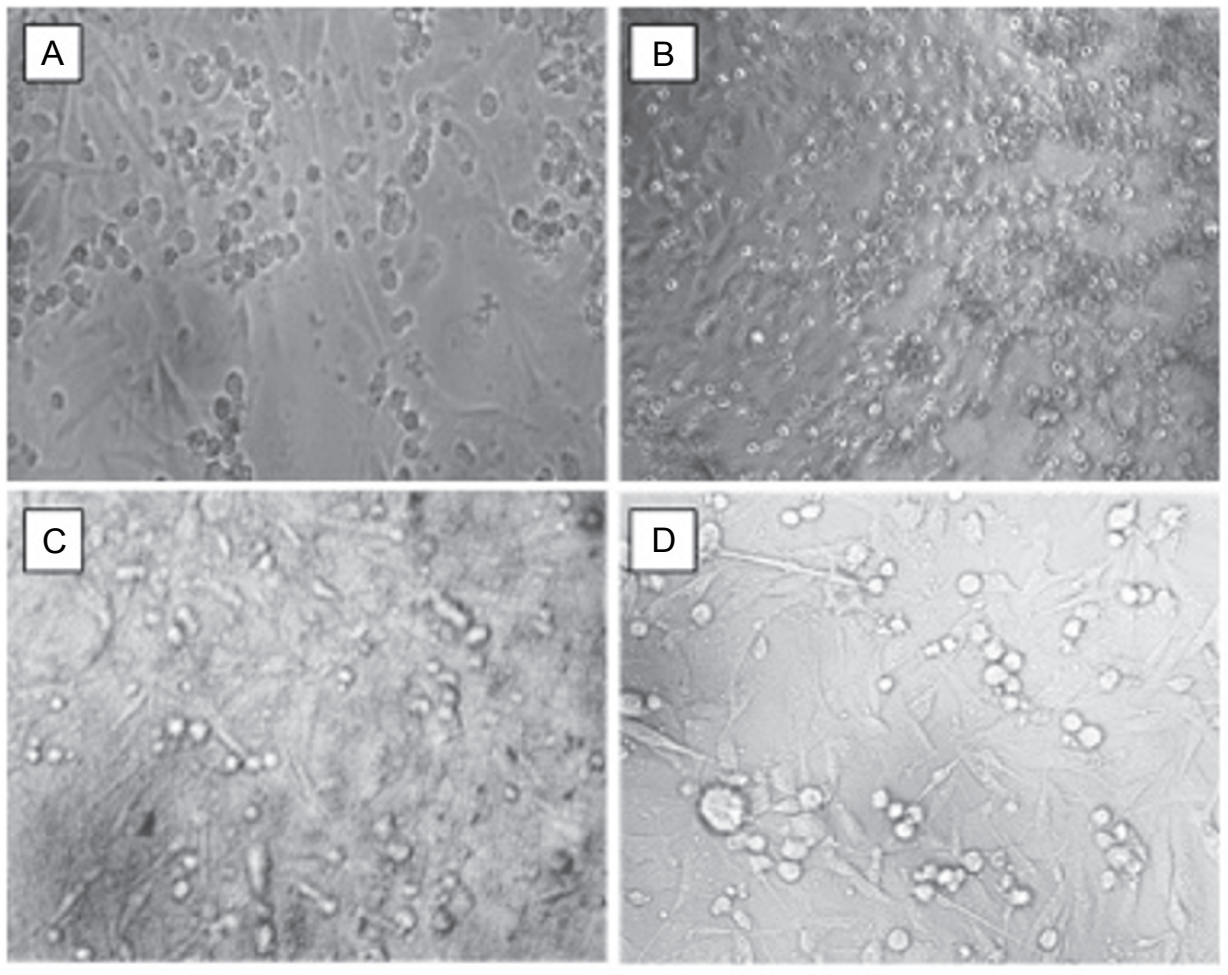
Cell biology studies of cells treated with A) control, B) doxorubicin, C) dichloromethane extract, D) ethyl acetate extract at their IC50 values (the images were taken at 10× magnification)

Subsequently, cells were exposed to the IC_50_ concentrations of doxorubicin, dichloromethane, and ethyl acetate extracts and analyzed for morphological changes under a phasecontrast microscope. Cells treated with medium alone served as the control. The observations revealed that cells treated with dichloromethane and ethyl acetate extracts of *L. aspera* exhibited a round morphology, whereas, the control group and the doxorubicinreated group did not show any change in morphology. Moreover, more detached cells were observed in the extracttreated groups (Fig. 9).

The current observations align with earlier studies conducted on *L. aspera*. Studies by Jayanthi et al. (2020) demonstrated that the ethanol extract from *L. aspera* flowers induced cytotoxicity in *Ehrlich ascites* carcinoma cells with an IC_50_ of 102.42 μg/ml under *in vitro* conditions. Similarly, reports by Augustine et al. (2014) showed that the ethyl acetate extract of *L. aspera* leaves induced cell death in Daltons Ascites Lymphoma (DAL) cells at an IC_50_ of 29.4 μg/ml.

**Fig. 10:**
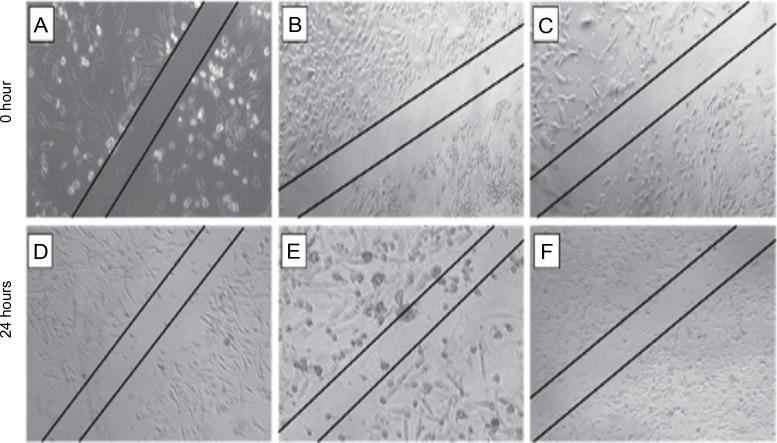
Cell migration analysis of *L. aspera* extracts; the figure shows A), B) cells that serves as control at 0 and 24 h, C), D) dichloromethane extract treated cells at 0 and 24 h, E) and F) ethyl acetate extract treated cells at 0 and 24 h; migration of cells inside the wound is observed at 24 h (images were taken at 5× magnification)

**Fig. 11:**
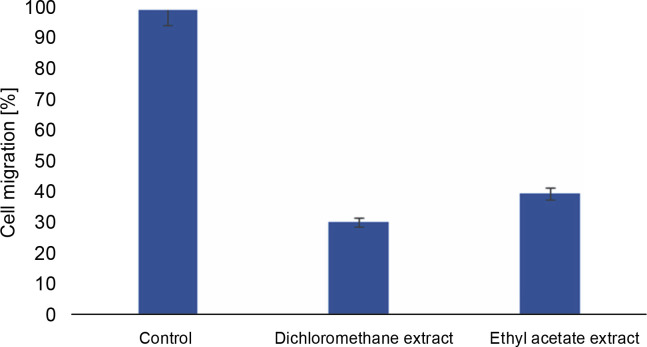
Antimigratory effect of *L. aspera* extracts; the percentage of cells migrated in treated cells is less compared to control (the values show mean ± SD)

The scratch assay was carried out to evaluate the effect of *L. aspera* extracts on inhibiting the migration of MDA-MB-231 cells under in vitro conditions. Cell migration plays a pivotal role in embryogenesis, wound healing, tissue development, and cancer metastasis (Olson et al., 2018). Understanding the mechanisms of cell migration is crucial for developing new therapeutics to control tumor invasion. The results of the scratch assay revealed that the number of cells migrating into the wound region was lower in cells treated with *L. aspera* extracts compared to the control group (Fig. 10). The percentage of cells migrating into the wound region in the control group was set as 100%. Based on this, the percentage of cells migrating into the wound was determined as 30.6% in the dichloromethane extract-treated group, whereas the percentage of migrated cells was 40% in the ethyl acetate extract-treated group (Fig. 11). The current observation indicates that the dichloromethane extract of *L. aspera* was more effective in preventing cell invasion compared to the ethyl acetate extract. Earlier studies have demonstrated that the methanol extract of *L. aspera* inhibited the migration of prostate cancer cells (PC3) at 0.25 mg/ml within 30 h (Mohan et al., 2017). In conclusion, it can be stated that dichloromethane and ethyl acetate extracts of *L. aspera* exhibit cell migration inhibitory properties.

## Conclusion

Based on the current study, it can be concluded that both dichloromethane and ethyl acetate extracts of *L. aspera* contain active phytochemical agents. These phytochemical compounds may contribute to the observed antiproliferative and anti-migratory activities of the plant extracts, in MDA-MB-231 cell tests. *L. aspera* could be a promising source for the development of therapeutic agents against triple-negative breast cancer. However, the specific compounds responsible for the antiproliferative and antimigratory activities have not been identified. Therefore, further studies are needed to identify and characterize these active compounds, providing insights into the effective management of TNBC.

## Supplementary Material

Phytochemical characterization by GC-MS and *in vitro* evaluation of antiproliferative and antimigratory studies of *Leucas aspera* leaf extracts on MDA-MB-231 cell line

## Data Availability

The data used and analyzed during the current study are available from the corresponding author upon reasonable request.
