# Supplementary material for: Phytochemical characterization by GC-MS and in vitro evaluation of antiproliferative and antimigratory studies of Leucas aspera leaf extracts on MDA-MB-231 cell line
Source: BioTechnologia (Pozn). 2024 Mar 29;105(1):55–68. doi: 10.5114/bta.2024.135642 (PMC11020152; doi:10.5114/bta.2024.135642)
Supplement: Phytochemical characterization by GC-MS and in vitro evaluation of antiproliferative and antimigratory studies of Leucas aspera leaf extracts on MDA-MB-231 cell line [file BTA-105-1-52457-s001.pdf]

BioTechnologia vol. 105 (1) • 2024
Journal of Biotechnology, Computational Biology and Bionanotechnology SUPPLEMENTARY MATERIALS
https://doi.org/10.5114/bta.2024.135642
CC BY 4.0

Phytochemical characterization by GC-MS and *in vitro* evaluation of antiproliferative and antimigratory studies of *Leucas aspera* leaf extracts on MDA-MB-231 cell line

FAZEELA MAHABOOB BEGUM S.M. *, MEGASRI SANKARRAM
B.S. Abdur Rahman Crescent Institute of Science and Technology, Vandalur, Tamil Nadu, India

Received: 24 May 2023; revised: 20 December 2023; accepted: 22 December 2023

Abstract

Breast cancer is the most recurrently identified and one of women's prominent causes of death. Currently, researchers have turned their focus on natural chemicals from synthetic chemicals due to their environmental, economic, and health benefits. Considering this, the medicinal plant *Leucas aspera* was chosen for the current study. The aim of this study was to isolate and characterize secondary metabolites from *L. aspera* and determine the antiproliferative and antimigratory activities in the MDA-MB-231 cell line under *in vitro* conditions. Phytochemicals from *L. aspera* were isolated through sequential extraction using hexane, dichloromethane, and ethyl acetate. These extracts were qualitatively screened, subjected to FT-IR, and analyzed using GC-MS. The antiproliferative activity was determined through the MTT assay. Scratch assay was utilized to determine the antimigratory activity of the plant extracts. The phytochemical analysis revealed the presence of steroids, alkaloids, phenols, flavonoids, galactose, tannins, saponins, and amino acids in the extracts. The results of the cell viability assay indicated that the crude dichloromethane and ethyl acetate extracts inhibited cell proliferation, with inhibitory concentrations of 5 and 3 μg/ml, respectively. In contrast, the crude hexane extract did not exhibit any cytotoxicity. Furthermore, the scratch assay results showed that the plant extracts had cell migration inhibitory properties. The outcomes of the current study conclude that *L. aspera* possesses active therapeutic agents with strong anticancer potential, effectively impeding the proliferation and invasion of MDA-MB-231. Further studies are needed to identify the potential active agents that contribute to these activities.

Key words: cytotoxic activity, MDA-MB-231, breast cancer, *Leucas aspera*, anti-migratory efficiency

* Corresponding author: B.S. Abdur Rahman Crescent Institute of Science and Technology, Vandalur, Tamil Nadu, India;
e-mail: fazeelabegum@crescent.education

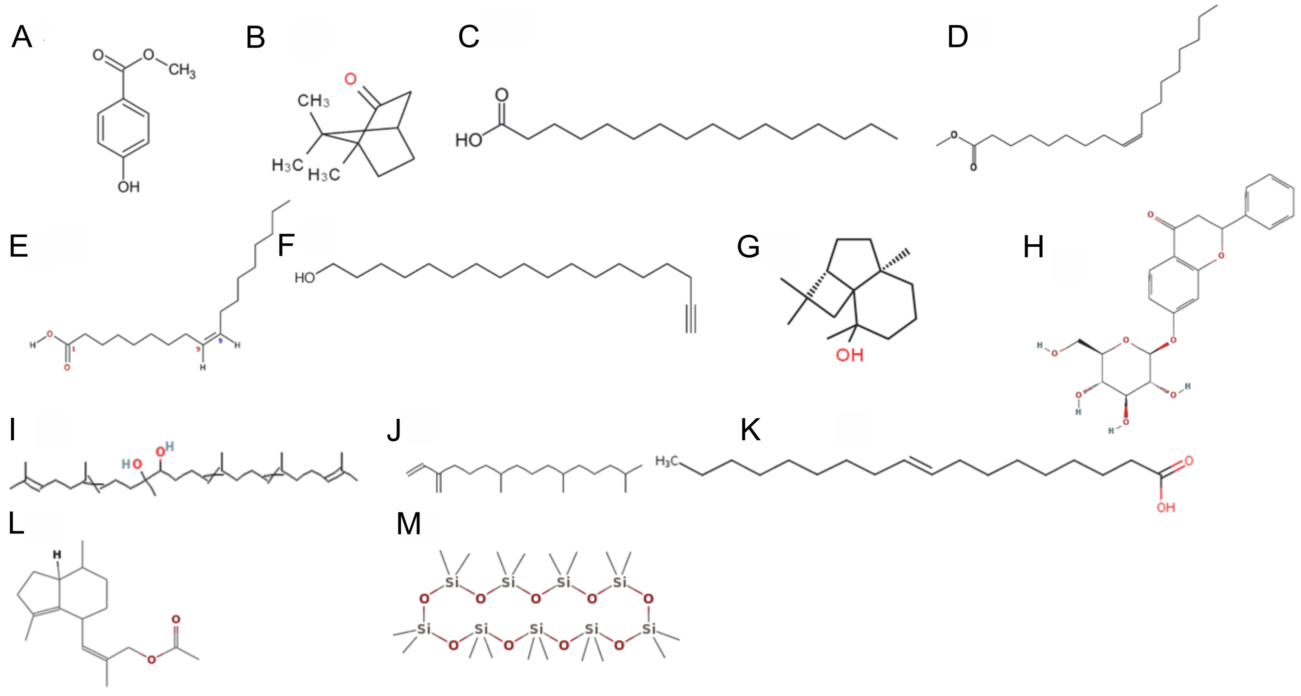

Fig. S1. Structures of compounds identified in dichloromethane extract of *L. aspera*. A) methylparaben, B) bicyclo [2.2.1] heptan-2-one, 5-bromo-1,7,7-trimethyl-, C) n-hexadecanoic acid, D) 6-octadecenoic acid, methyl ester, (Z)-, E) oleic acid, F) 17-octadecen-14-yn-1-ol, G) 2,2,4a,7a-tetramethyldecahydro-1H-cyclobuta[e]inden-5-ol, H) flavone 4'-oh,5-oh,7-di-o-glucoside, I) 2,6,10,15,19,23-hexamethyl-tetracosa-2,10,14,18,22-pentaene-6,7-diol, J) neophytadiene, K) octadecanoic acid, L) (z)-valerenyl acetate, M) cyclononasiloxane (the structures of compounds were downloaded from PubChem)

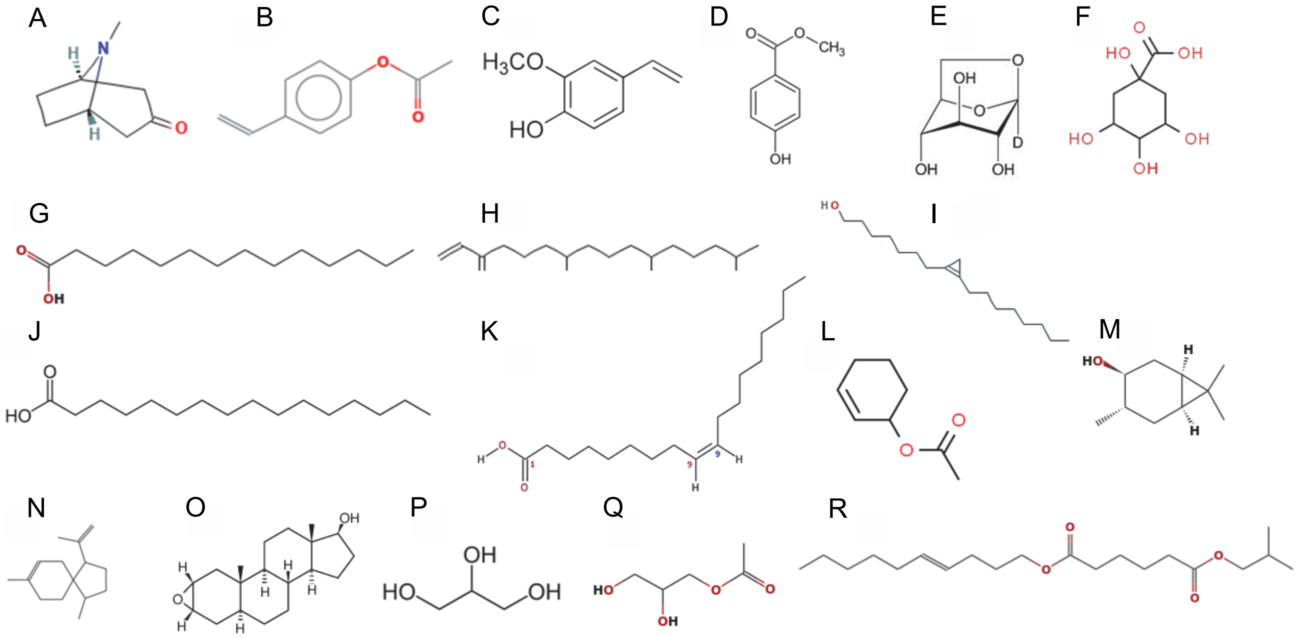

Fig. S2. Structures of compounds identified in ethyl acetate extract of *L. aspera*; A) 8-azabicyclo[3.2.1]oct-6-en-3-one, 8-methyl B) phenol, 4-ethenyl-, acetate, C) 2-methoxy-4-vinyl phenol, D) methyparaben, E) beta-D-glucopyranose, 1,6-anhydro, F) 1,3,4,5-tetra-hydroxycyclohexanecarboxylic acid, G) tetradecanoic acid, H) neophytadiene, I) 2-octylcyclopropene-1-heptanol, J) n-hexadecanoic acid, K) oleic acid, L) (cyclohex-2-enyl)acetic acid, M) bicyclo[4.1.0]heptan-3-ol, 3,7,7-trimethyl-, N) spiro[4.5]decan-7-one, 1,8-di-methyl-8,9-epoxy-4-isopropyl-, O) androstan-17-ol, 2,3-epoxy-, (2.alpha.,3.alpha.,5.alpha.,17.beta.), P) glycerin, Q) 1,2,3-propanetriol, 1-acetate, R) adipic acid, dec-4-enyl isobutyl ester (the structures of compounds were downloaded from PubChem)
